# T125. INCIDENCE OF TREATMENT RESISTANCE SCHIZOPHRENIA IN A COMMUNITY SAMPLE USING THE TRRIP CONCENSUS

**DOI:** 10.1093/schbul/sby016.401

**Published:** 2018-04-01

**Authors:** Dan Joyce, Rodrigo Bressan, Sukhi Shergill

**Affiliations:** 1 Institute of Psychiatry, King’s College; 2 Universidade Federal de Sao Paulo - UNIFESP; 3 Cognition Schizophrenia and Imaging (CSI) Lab

## Abstract

**Background:**

Estimates of treatment resistant schizophrenia (TRS) vary due to lack of consensus definition. The Treatment Response and Resistance in Psychosis (TRRIP) consensus provides a rigorous prospective definition for TRS, but has not yet been applied to data. We provide the first prospective estimate of the incidence of TRS in a large community cohort using TRRIP.

**Methods:**

The publicly available CATIE (Clinical Antipsychotic Trials of Intervention Effectiveness) data were mined using bespoke implementations of algorithms that operationalise the minimum TRRIP concensus criteria. Survival curves for transition to treatment resistance status (versus treatment responsive and censoring) were estimated. Inferential methods were used to establish baseline patient characteristics that are associated with TRS. Machine learning methods were also applied to estimate patient-level prediction of TRS status from baseline data.

**Results:**

1369 patients were included in the analysis, with 992 patients at risk for developing TRS at baseline. A total of 48 cases of TRS were identified, yielding a crude incidence of 36.2 cases per 1000 person years. There were no strong associations with baseline demographics or clinical state at enrolment to the trial and the predictive modelling failed to identify any patient-level predictor of future TRS.

**Discussion:**

The CATIE trial protocol excluded patients with retrospective evidence of TRS, however, prospectively applying the TRRIP consensus revealed that there were patients with TRS in the cohort. Our results suggest a small incidence, and that baseline clinical and demographic data is not a robust predictor of future resistance status. Analysis of individual TRRIP criteria reveals a significant unmet need for patients with poor treatment response, but who do not meet criteria for TRS, particularly in social and occupational functioning.

